# Firefighters’ medical use and Korean Medicine experience in Korea: A qualitative study protocol

**DOI:** 10.1371/journal.pone.0300532

**Published:** 2024-03-25

**Authors:** Jisu Ha, Inae Youn, Yuri Han, Jinwon Kim, Sunjoong Kim, Hanbit Jin, Jung Won Kang, Jungtae Leem

**Affiliations:** 1 Department of Acupuncture and Moxibustion, National Medical Center, Seoul, Republic of Korea; 2 College of AI convergence, Donggkuk University, Seoul, Republic of Korea; 3 Department of Internal Medicine of Korean Medicine, National Medical Center, Seoul, Republic of Korea; 4 Department of Diagnostics, College of Korean Medicine, Wonkwang University, Iksan, Jeollabuk-do, Republic of Korea; 5 Department of Acupuncture and Moxibustion, College of Korean Medicine, Kyung Hee University, Seoul, Republic of Korea; 6 Research Center of Traditional Korean Medicine, Wonkwang University, Iksan, Jeollabuk-do, Republic of Korea; PLOS: Public Library of Science, UNITED KINGDOM

## Abstract

**Introduction:**

Firefighters, compared to other occupational groups, are exposed more frequently in their working environment not only to physical issues, such as musculoskeletal disease, respiratory disease, and burns but also to mental health issues, such as PTSD and depression. Specifically, Korean firefighters experience significantly higher rates of work-related injuries compared to those in other countries. Recent statistics from the Korea National Fire Agency indicate a steady increase in the number of firefighting work-related injuries. However, there is a shortage of measures in place to address these issues. This study aims to investigate the health needs, overall healthcare usage, and unmet needs of firefighters in Korea. We also aim to investigate, through in-depth interviews, perceptions and hindering factors for integrative medicine approaches to fulfilling unmet needs.

**Method:**

This study was conducted in accordance with the consolidated criteria for reporting qualitative research. Convenience and snowball sampling methods will be used to recruit firefighters to participate in the study, and interviews will be conducted using a semi-structured interview guide. The data will be analyzed in four stages using the qualitative analysis method of Krippendorff.

**Discussion:**

In this study, we examine the state of health issues and healthcare usage among Korean firefighters and investigate their perceptions of and needs for integrative medicine. In this way, we aim to explore how integrative medicine and Korean medicine approaches could improve and assist healthcare services for firefighters. Furthermore, our findings will provide policymakers and healthcare providers with the necessary basic information to develop integrative medicine systems suited to firefighters.

## Introduction

Firefighters are exposed to hazardous environments that can cause both physical harm and psychological trauma by the nature of their jobs [1, 2]. In Korea, firefighters are civil servants who work at fire service facilities, including fire stations, fire and safety departments, and the Korea National Fire Agency, performing various fire service-related tasks, including fire prevention, fire safety monitoring, firefighting, and rescue [3]. Due to the nature of their work, firefighters are at risk of various diseases due to exposure to environmental risk factors and hazards [4]. Also, the work-related injuries rate among Korean firefighters is noticeably higher than that of firefighters in other countries. According to recent statistics from the Korea National Fire Agency, the number of injuries related to firefighting work has steadily increased over the past 10 years [5, 6]. First, respiratory diseases, such as pneumonia, asthma, and chronic obstructive pulmonary disease (COPD), are common among firefighters, due to exposure to smoke and other harmful substances [7]. Second, there is a risk of skin injury due to burns and exposure to chemical substances [8]. Third, carrying equipment, performing rescues, and other physically challenging tasks lead to a high incidence of spine and joint disorders [9]. Finally, firefighters can experience heavy stress at accident sites, causing emotional problems such as depression, anxiety disorder, and post-traumatic stress disorder (PTSD) [10].

In the United States, which has good welfare for firefighters, standard guidelines (NFPA 1583) from the National Fire Protection Association (NFPA) propose more systematic wellness/fitness programs for the safety, health management, and welfare of firefighters [11]. Meanwhile, they provide care programs for mental health issues like PTSD through peer support, where firefighters with expertise in psychiatric counseling provide counseling directly. However, even though the importance of health improvements for firefights has been emphasized, and there have been studies on the effects of health management programs, these programs are still not being implemented at over 70% of fire stations, leading to a shortage of actual care with regard to health management for firefighters [12].

In Korea, the National Fire Agency runs various mental health programs for firefighters, including managing work stress, managing PTSD, improving motivation for learning, and self-management [13]. However, the programs are of poor quality and are not systematic [14]. According to a study on the state of health management programs for emergency responders, although most facilities (88.3%) were running health management programs involving physical training facilities and special lectures, satisfaction was not high [15]. Thus, although various efforts are being made at the national level to manage the physical and mental health of firefighters, there are still unmet needs, especially for pain, rehabilitation, burns, and psychological issues, such as PTSD [16]. To resolve the unmet needs of firefighters regarding health problems, it is necessary to determine these unmet needs and devise measures to fulfill them.

In East Asia, various types of traditional medicine are used for health issues [17], and in Korea, civil servants also utilize Korean Medicine(KM) for health issues [18]. In South Korea’s binary Western/Korean healthcare system, because KM treatments are included in the National Health Insurance Service, accessibility is relatively high, and for patients’ convenience and effective treatment, integrative medicine is often provided as a form of collaborative care [19, 20]. Several systematic literature reviews have reported positive effects of KM on conditions commonly experienced by firefighters, including musculoskeletal pain [21], postoperative pain management [22], respiratory problems [23], digestive problems [24], headache [25], depression [26], and PTSD [27]. Therefore, KM can play a role in supporting their health and well-being in several ways.

However, studies on the unmet health needs of firefighters or the potential role of KM in these issues are lacking. Moreover, there is an insufficient in-depth understanding of Korean firefighters’ overall healthcare utilization; thus, it remains unclear what the expectations and obstacles are for integrative medical approaches. Therefore, we plan to conduct a study aimed at investigating Korean firefighters’ health needs, overall healthcare usage, and unmet needs to generate useful data supporting personalized improvements in healthcare services for Korean firefighters. Our findings will provide policymakers and healthcare providers with the necessary basic information to develop integrative medicine systems suited to firefighters.

## Materials and methods

### Objectives

The primary objective of this study is to gain an in-depth understanding of the unmet needs of Korean firefighters regarding healthcare utilization, from the firefighters’ perspective.

The secondary objective is to examine what experiences Korean firefighters have of integrative medicine, obstacles to its use, and how it could help with their unmet needs.

### Study design

In this qualitative study, we use content analysis to explore overall content on the state of healthcare utilization by Korean firefighters and their perceptions and needs regarding KM. This study will be performed in accordance with the consolidated criteria for reporting qualitative research (COREQ) [28]. We provide COREQ checklist (S1 Checklist), the Korean version of the full protocol (S1 File), and English version of full protocol (S2 File).

### Sample size

To ensure the validity of this study, we will use a sample size of 20 persons, which is the suggested sample size for qualitative research [29].

However, after interviewing 20 persons, the researchers can perform additional interviews if they feel the data is not yet saturated.

Saturation is a criterion for stopping data collection, referring to the state where no new data is being discovered from new participants, and so no further qualitative interviews are required.

### Participant recruitment

Recruitment is scheduled to commence on October 4, 2023, and conclude on December 31, 2024. Participants will be recruited using two different methods. First, using convenience sampling, with cooperation from the Korea National Fire Agency, we will recruit participants via an official notice posted to the internal intranet. We will then use snowball sampling to recruit other participants. This is a non-probability sampling method, where other participants are recruited through referral by previous participants; this is an effective method for studying relatively inaccessible populations [30]. The inclusion criteria are Korean firefighters aged 19–65 years who voluntarily consent to participate in the study. Considering that 19 years old marks the legal age of adulthood in South Korea, we recruited participants ranging from 19–65 years old to gain a comprehensive understanding of adulthood. And we include retirees to get more opinions. To hear diverse opinions from the firefighters, we will consider sex, age, job family, region, and years of service. We will explain the objectives and methods of the study, and any potential benefits or disadvantages to participants, and inform them that audio or video of the interview content will be recorded and transcribed. After being thoroughly informed, firefighters who decide to voluntarily participate in the study will complete the written consent form. Participants can be excluded if the researchers judge that it will be difficult for them to participate in the interviews.

### Data collection

We plan to hold the interviews between October 2023 and December 2024. The researchers will collect data through semi-structured interviews using either one-to-one in-depth interviews or focus group interviews (Table 1). This is one of the most common methods of data collection in qualitative studies, and because of its flexible structure, researchers can collect more information from the interview participants, and the participants can also freely express their own opinions [31, 32]. All interviews between researchers and participants will be conducted in Korean. The interviews will last 60–150 min per session, and each individual could participate in up to five interview sessions. The interviews will be conducted in a private space, where the participants can feel comfortable. If in-person interviews are not possible, remote interviews will be held using Zoom. Participants will be asked about their everyday health status, their reason for visiting the hospital, and their experiences and perceptions of KM treatment. With the participants’ consent, audio or video recordings will be made of the interview content. Non-verbal communication during the interview, such as body language or gestures, can be noted by the interviewer in person. In accordance with research ethics regulations, participants will be given appropriate compensation of 100,000 KRW per interview session for participating in the study.

**Table 1:** Interview guide.

Research questions	Detail questions
Overall experiences of healthcare utilization	1. How is your everyday health? 2. Is there anything you feel is a health problem? 3. What treatment have you received to resolve your health problems? Have you received Korean Medicine treatment? 4. If you have not received treatment or management, why not?
Perceptions and experiences of education or programs provided by the Korea National Fire Agency	1. Do you know what education or programs are provided? 2. Have you received any help? 3. How do you feel about your experiences? 4. If you were the Korea National Fire Agency, what education or programs would you offer?
Perceptions and experiences of Korean Medicine	1. How do you typically feel about Korean Medicine? 2. Are you, or are you not in favor of Korean Medicine? Why? 3. Do you have any experience of Korean Medicine? If yes, what symptoms have you received treatment for? 4. Have your perceptions changed since receiving Korean Medicine treatment? 5. Were you satisfied after receiving Korean Medicine treatment? If you were satisfied/dissatisfied, please explain why? 6. If you have not experience Korean Medicine, why not? 7. What aspects of Korean Medicine do you think should be improved?
Summary of interview content	After summarizing the above interview contents, we will check we have properly understood the participant’s intentions.
Additional content	Is there anything more you would like to talk about?

### Data analysis

The collected data will be analyzed using content analysis, which is a method of analysis in qualitative research, with the aim of understanding phenomena [33]. Content analysis is a valid method that enables specific inferences, based on systematic coding rules, from expansive data collected through interviews [34].

The collected data will be analyzed according to the following procedures:

In the first stage, the researchers will try to understand the overall data. While repeatedly reading the transcribed interview content, the researchers will review, from an objective perspective, which of the participants’ experiences of healthcare utilization are related to Korean firefighters’ perceptions and needs concerning integrative medicine.

The second stage is the process of finding significant statements. Here, the researchers will make sentences from the participants’ interview content, dividing these into analytic units of words, phrases, and sentences. the researchers will then discover significant statements related to Korean firefighters’ healthcare utilization and integrative healthcare and will reconstruct the meaning of these statements.

The third stage is categorization. The researchers will make concepts from the significant statements and combine mutually related or similar concepts to create categories.

In the final stage, the researchers will reorganize the constructed categories into social, personal, and cognitive dimensions.

Before the start of the study, researchers are female doctors of KM (IA, JH) who have learned qualitative research methodology through lectures and writings by qualitative research experts and oversee a research methods and analysis course. All interviews will be discussed by multiple researchers to minimize individual bias. To improve the reliability of the coding process and reflect diverse perspectives, an additional two researchers other than the interviewers will independently evaluate the results through meetings and verify the suitability of the significant statements. One of these additional researchers is a qualitative research expert, not a doctor of KM, and is currently participating in qualitative research education and several qualitative research projects. The additional researcher has PhD in KM and is actively involved in various fields of research, including both qualitative and clinical research. In addition, to increase the reliability and validity of the results, an independent researcher will validate the coding through triangulation.

### Patients’ involvement

In this study, patients will be involved in the whole research procedure, allowing us to collect more abundant, in-depth data. During the interviews, the interview sheet can be adjusted depending on the participants’ experience and preferences. During the interviews, we will communicate with the participants about methods of effectively publicizing our research results and improving their usability.

### Ethical considerations

This study was approved by the National Medical Center’s institutional review board (August 24, 2023; NMC-2303-08-089), and the protocol was submitted to the Clinical Research Information Service (CRIS; KCT0009108). Before participating in the study, participants will be provided with a written consent form explaining the study purpose, protection of personal information and privacy, risks and benefits, participation, and the right to withdraw from the study at any time. Participants will then sign the form. To protect the participants’ anonymity, each participant will be assigned an identification code. Personal information collected to pay compensation to the participants will be managed in accordance with the Personal Information Protection Act and destroyed immediately after payment. In accordance with the Bioethics and Safety Act, records related to the study will be stored for 3 years after completion of the study and then destroyed.

## Discussion

This study aimed to examine, through interviews, Korean firefighters’ health issues and the state of their healthcare utilization to solve these issues to explore how integrative healthcare approaches and KM approaches could help. In this way, we intend to investigate the Korean firefighters’ perceptions and needs with regard to integrative healthcare.

Due to their working environment, firefighters often have to perform excessive work in unpredictable situations, or work requiring too much physical exertion, which exposes them to a high risk of musculoskeletal pain, sprains, and strains [35]. In particular, many firefighters complain of chronic lower back pain, at a ratio higher than general labor occupations [36]. Irregular lifestyles due to their working patterns often lead to problems such as dyspepsia and insomnia [37, 38]. Moreover, firefighters frequently experience potentially psychologically traumatic events, leading to a high prevalence of various psychiatric problems, including PTSD, anxiety, and depression [39].

At the government of Korea, various health management programs have been developed for firefighters experiencing such difficulties [40, 41], and support is provided by referral to hospitals, allowing firefighters to receive regular examinations [42]. In addition, there are plans to construct a National Fire Hospital, for expert treatment, management, and research of diseases that commonly affect firefighters [43].

According to statistics [44], musculoskeletal pain, headaches, and functional dyspepsia frequently experienced by firefighters are all complaints commonly treated at KM institutions; thus, many patients are seeking KM treatments for these symptoms. In addition, multiple studies have demonstrated that KM is effective for treating these conditions. KM methods, including acupuncture, moxibustion, and *chuna* manual therapy, are effective at reducing pain in musculoskeletal disorders [45–50]. Several studies have shown that acupuncture is effective for short- and long-term alleviation of various types of musculoskeletal pain. In addition, acupuncture and herbal medicine ameliorate symptoms and help improve the quality of life in patients with respiratory and digestive problems [51–54]. A systematic review reported that when KM and Western medicine were combined in the treatment of COPD, patients showed improved symptoms and quality of life, demonstrating a stronger effect than Western medicine alone [55, 56].

A prospective study reported that acupuncture treatment was effective at alleviating pain and reducing analgesic (opioids) use in burn treatment [57], and another case series has been published showing improvements without surgery when burns requiring skin graft were treated with acupuncture and ointment [58].

Additionally, clinical are constantly being published showing the effectiveness of acupuncture [59], herbal medicine [60, 61], and auricular acupuncture [62] on psychiatric problems, such as PTSD, depression, and anxiety. In the cases of PTSD, depression, panic disorder, and insomnia following a disaster, auricular acupuncture improves quality of life, relieves anxiety, and reduces symptoms of depression [63]. Especially for improving and stabilizing symptoms of depression, herbal medicine shows an equivalent effect to antidepressants with fewer adverse effects, and when taken together with antidepressants, reduces adverse effects [64, 65].

Acupuncture modulates and transmits pain sensations via nerve conduction, releasing endogenous substances like beta-endorphin, enkephalin, and dynorphin, which act to inhibit pain sensation [66, 67]. Auricular acupuncture influences the vagal activity of autonomic functions across cardiovascular, respiratory, and gastrointestinal systems, leading to its application in pain relief, anxiety treatment, and sleep quality enhancement [68, 69].

Various clinical studies and systematic reviews have given evidence of the effectiveness of KM treatment for diseases that are prevalent among firefighters. However, studies on the use of KM treatment for firefighters are lacking. Most studies on firefighters in Korea and overseas aimed to investigate their health status. For example, there has been a qualitative study on firefighters’ experiences of PTSD due to work [70], experiences and coping with emotional labor [71], a questionnaire study [72, 73], and a prospective study [74] on fatigue, insomnia, and psychiatric health problems due to work stress, and a study analyzing work-related injuries [75]. However, there have been no in-depth studies on firefighters’ experiences of KM. Therefore, our study will help ascertain firefighters’ overall healthcare utilization and unmet health needs, helping provide an in-depth understanding of the KM treatments required by firefighters.

Thus, this study provided insights into directions for the development of the upcoming Korea National Fire Hospital based on the limitations of Korean firefighters’ healthcare utilization, and their experiences and perceptions of KM. Furthermore, the results can be referenced when providing integrative medicine approach in hospitals specializing in musculoskeletal pain, PTSD, and burns in other countries. Our findings may also be helpful in providing for public healthcare institutions, including the National Police Hospital and the National Traffic Injury Rehabilitation Hospital. This study has a limitation. Although we intended to recruit firefighters nationwide, we could not recruit participants evenly across the country; thus, it is difficult to generalize the sample to the whole population of firefighters. In this study, the inclusion of KM practitioners could lead to the results reflecting researchers’ favorable bias towards KM. However, their involvement also offers an advantage, enabling more in-depth research due to their better understanding of KM treatment.

In a follow-up study, we intend to expand the multidisciplinary research by including more diverse healthcare occupational groups.

### Dissemination

We plan to broadly publicize the results of this study through academic journals, newspapers, academic seminars, and conference presentations. Moreover, we additional intend to expand upon the results through public health policy development meetings based on our findings. These results will be published at annual online and offline academic seminars for KM practitioners. In this way, we anticipate that our study will be widely publicized in academia and policy-related fields, thus contributing to exchange and policymaking among experts in these fields.

## Supporting information

S1 Checklist COREQ checklist. (PDF)

S1 File Study protocol for IRB Korean. (DOCX)

S2 File Study protocol for IRB English. (DOCX)
