# Supplementary material for: Firefighters’ medical use and Korean Medicine experience in Korea: A qualitative study protocol
Source: PLoS One. 2024 Mar 25;19(3):e0300532. doi: 10.1371/journal.pone.0300532 (PMC10962829; doi:10.1371/journal.pone.0300532)
Supplement: S1 File — (DOCX) [file pone.0300532.s002.docx]

**연구계획서**

**연구 제목 국문: 소방공무원들의 전반적인 의료이용 및 한의치료에 대한 인식과 수요조사를 위한 질적연구**

**연구 제목 영문: Qualitative Study on the Perception and Demand for Overall Medical Utilization and Korean Medicine Treatment among Firefighters**

**Version No: Ver.1.2.**

**책임연구자 소속: 국립중앙의료원 한방진료부**

**책임연구자 이름: 윤인애**

**학위논문 여부: 예 ( o ) / 아니오 ( ) 연구계획서**

1. **연구 제목**

소방공무원들의 전반적인 의료이용에 대한 경험 및 한의치료에 대한 인식/수요에 대한 질적 연구

1. **연구의 실시기관 명칭 및 주소**

### 국립중앙의료원 서울시 중구 을지로 245(을지로 6가 18-79)

1. **연구책임자 및 공동연구자 성명 및 직명**
2. **연구책임자**

### 윤인애 (국립중앙의료원 한방진료부침구과 과장)

1. **공동연구자**

### 임정태 (원광대학교 한의과대학 교수)

김진원 (국립중앙의료원 한방진료부 부장)

### 하지수 (국립중앙의료원 침구과 전공의)

### 김선중 (국립중앙의료원 침구과 전공의)

1. **연구비 지원기관 명칭 및 주소**

### 한국연구재단

대전청사: 대전광역시 유성구 가정로 201

서울청사: 서울특별시 서초구 헌릉로 25

1. **예상 연구기간**

### IRB 승인일 ~ 2024년 12월 31일까지

1. **연구 대상 군**

### 전국 소방공무원

1. **연구의 배경 및 목적**
2. **연구 배경**

### □ 국립소방병원의 설립 배경

### - 소방공무원은 직무의 특성상 늘 재난현장에서 위험하고 참혹, 충격적인 상황에 반복적으로 노출됨에 따라 부상과 트라우마 등 정신적 고통에 시달리고 있어 체계적인 진료와 연구, 관리가 필요함.

### - 이에 소방공무원 관련 질환을 전문적으로 치료, 관리, 연구하는 시설 및 의료진이 필요한 상황.

### - <국립소방병원의 설립 및 운영에 관한 법률>이 제정 및 시행되며, 소방공무원의 진료와 특수한 근무 환경에 따른 건강유해인자 분석 및 질병 연구를 통하여 체계적인 소방공무원의 건강관리 및 교육을 위해 국립소방병원을 설립하게 됨.

### □ 국립소방병원 내 한의과 개설 및 운영의 필요성

### ○ 국가 공공의료기관의 기능과 역할 수행

### - 공공의료기관으로서의 국민의 의료선택권과 접근성을 보장하기 위해 설치 필요.

### ○ 소방공무원의 건강상의 문제와 한의의료가 밀접한 연관성을 가짐.

### - 소방공무원 10개 유병률 중 (건강보험)한의의료 다빈도 질환에서 6개 질환이 공통.

### - 소방공무원에서 유병률이 높은 근골격계 질환은 한의의료의 대표적인 치료 분야로 한의의료 다빈도 질환 순위 50위 중 18개가 포함되어 있음.

### 2) 선행 연구

### □ 소방공무원의 특수한 근무환경에 따른 질병 및 상해와 관련한 한의치료 관련 선행 연구

### - 근골격계 통증과 관련한 한의치료

### 미국에서 두 번째로 큰 의학 그룹인 American College of Physicians의 급성, 아급성, 만성요통에 대한 비침습적 진료지침에서 급성/아급성 요통에 침치료(low-quality evidence), 만성 요통에 침치료(moderate-quality evidence)와 추나치료(spinal manipulation, low-quality evidence)를 강력한 등급(Grade: Strong)으로 권고함.

### - 외상 후 스트레스 장애 관련 한의치료

### 한국연구재단의 지원으로 여러 연구에 근거, 2022년 재난 트라우마의 한의사 진료매뉴얼이 출판됨.

### - 화상 관련 한의치료

### 피부이식이 필요할 정도의 깊은 화상을 입은 환자 4명을 수술 없이 한약재 연고와 침치료로 크게 개선시킨 증례보고가 해외 화상치료 학술지에 발표됨.

### - 호흡기계 관련 연구

### 만성폐쇄성폐질환에 대한 침치료 관련 문헌고찰에서 33개의 연구를 고찰한 결과 경혈지압이 호흡곤란, 삶의 질, 불안에 효과가 있었다는 결과.

### 3) 연구의 필요성

### □ 해외 사례 (미국의 재향군인을 위한 공공의료기관)

### ○ 미국 재향 군인에 대한 한의치료 관련 선행 연구

### - 소방공무원과 같이 특수한 근무환경에 따른 질병/상해를 겪는 재향군인에 대한 한의치료 임상연구 결과, 재향군인 PTSD, 재향군인 PTSD로 인한 불면, 재향군인의 통증에 한의치료 효과성 입증

### ○ 미국 재향 군인의 통합의학(한의치료 포함)에 대한 요구도 상승 및 의료서비스 확대

### - 만성 통증, 불안, 우울, PTSD, 중독 등에 대해 약물치료의 오남용(opioid crisis)에 대한 대안으로 통합의료에 대한 요구도가 높아지고 있는 실정.

### - 군인들에 대한 한의치료 요구도 연구 결과, 재향 군인들은 침치료 등에 대한 요구도가 높고, 더 많이 이용하고자 함(3346명을 대상으로 조사한 결과, 84% 넘는 군인이 통합의학을 이용한 경험).

### - 이에 미국에서는 재향군인을 위한 공공의료기관에서 한의의료서비스를 확대 제공하고자 함.

### ○ 또한 미국의 보훈보건청(VA, The Veterans Health Administration)는 2016년 법률제정을 통해 (2016 Comprehensive addiction and recovery Act) 침치료, 추나치료를 포함한 통합의학적 접근의 제공을 확대하도록 의무화함.

### □ 현재 2025년 6월 개원 예정인 국립소방병원 내 한의과 설치 미정인 상태

### - 상기한 바와 같이 소방공무원의 특수한 근무환경에 따른 질병 및 상해와 관련해서 한의의료서비스가 제공할 것이 많고, 그 효과에 대한 연구 결과도 많음.

### - 해외에서는 선호도 및 효과성을 고려하여 공공의료기관에 한의과 설치를 의무화하고 있는 반면에 국내에서는 세계 최고 수준의 한의치료 인력을 보유하고 있으나 신설하는 국립소방병원 내 한의과 설치계획이 없는 상황임.

### - 한의 공공의료 확대방안 연구에 의하면, 기존의 공공병원 내에 한의과 설치를 논의하는 것은 더욱 어려우며, 의료기관 설립이 완료되기 이전에 한의과 설치에 대한 논의를 하는 것이 보다 효율적이라고 함.

### - 따라서 소방공무원들에 대해 적정진료, 최선의 의료서비스를 제공하며 관련 연구를 수행해야 할 국립소방병원 관련하여 설립 임박한 현재 이 시점에서 한의과 설치에 대한 논의가 필요할 것으로 보임.

1. **연구 가설 및 목적**

□ 소방국립병원 내 한의과 설치의 타당성과 필요성에 대한 연구를 위해서, 소방공무원을 대상으로 한의치료에 대한 선호도, 요구도, 만족도와 전반적인 한의치료에 대한 인식 및 경험에 대해 알아보고자 함.

□ 나아가 본 연구의 결과를 공공병원 내 한의과 설치 또는 관련 정책의 근거자료로 활용하며, 후속 관련 사례의 모범적인 모델로 활용하고자 함.

1. **연구 방법**
2. **연구 대상 선정 기준 및 제외 기준**

**- 선정기준**:

- - - - 만 19세 이상 ~ 만65세 이하
      - 소방공무원으로서 현장 근무 중이거나 경험이 있는 사람
      - 위의 조건을 만족하며, 연구에 대한 충분한 설명을 듣고 자발적으로 연구참여에 동의한 사람
    - **제외기준:**
- 소방공무원 중 현장업무의 경험이 없는 자
- 연구자가 인터뷰 수행이 불가능하다고 판단할 경우

**- 중도탈락 기준:** 연구 대상자가 더 이상 연구 진행을 원하지 않는 경우

1. **연구대상자수**

- 소방공무원 대상 심층 면담 연구 대상자 수: 20명

- 이는 일대일 면담 대상자와 포커스그룹 인터뷰 대상자를 모두 포함한 수로, 일대일면담 대상자가 포커스그룹 인터뷰를 중복으로 한 경우 카운팅에서 제외함.

- 일대일 면담과 포커스그룹 인터뷰 각각의 인터뷰 대상자 수를 제한하지 않음.

1. **연구대상자 산출 근거**

- 연구 대상자 표집은 자료의 포화에 이르렀을 때 종료할 것이며, 연구 참여자수의 타당성 확보는 체험에 관한 질적 연구를 실시함에 있어 Dukes(1984)에 의하여 권고된 10~20명 전후를 만족하여 표집할 것이다. 이와 함께 소방공무원들을 대상으로 하는 질적 연구의 표집수에 근거하여 연구대상자 수 타당성을 확보할 것이다. 단, 20명의 환자를 인터뷰했음에도 불구하고 자료의 포화가 이뤄지지 않은 경우에는 추가적으로 인터뷰를 진행할 수 있다.

- 균형있는 시각을 얻기 위해 한의치료 경험이 있는 자와 없는 자의 모집 수를 동일하게 맞춘다.

- 포커스 그룹 인터뷰는 그룹 당 6~10명으로 구성되며, 일대일 면담 대상자 중에서 선정하거나 혹은 별도의 패널을 구성할 수 있다.

- 포화(saturation)는 새로운 패널에서 더 이상 새로운 이야기가 도출되지 않고, 기존의 패널에서 언급되었던 이야기가 반복되어서 정보가 포화상태에 이르고, 다음 패널에서의 질적 면담을 수행할 필요가 없는 상태를 말한다.

1. **연구 대상자 모집 방법 및 동의 과정**

### □ 연구대상자 모집

### - 대상자 모집은 2023년 10월 4일부터 2024년 12월 31일까지 진행할 예정이다.

### - 편의표집(Convenience Sampling)을 이용하여 소방청에 공문으로 협조 요청 후, 소방공무원 내부 인트라넷에 연구 광고물 게시 및 홈페이지 광고를 링크하여 참여자를 모집한다. IRB 승인 이후부터 게시하며 광고물에는 연구 제목 및 내용, 연구 참여자 기준, 면담 일시, 장소, 참여 시 얻게 되는 이익과 불이익, 연구자 연락처 등을 싣는다.

### - 또한, 모집한 대상자의 소개를 받는 눈덩이 표집(Snowball Sampling)을 통하여 연구대상자를 모집한다.

### □ 연구대상자 동의

### - 연구참여자 동의 과정 : 연구참여자가 연구에 적합한지 검토한다. 그 후, 연구책임자 또는 권한을 위임받은 연구담당자는 관련 법규 및 규정에서 요구되는 동의와 관련된 모든 사항을 포함한 연구참여자 설명서 및 동의서를 제시하고, 이 내용을 연구참여자 또는 법적 대리인에게 서면으로 설명한다.

### - 연구참여자가 시간, 장소의 문제로 대면 인터뷰가 어려울 경우, Zoom이나 Google meeting과 같은 화상회의프로그램을 통해 설명문을 보면서 연구자가 직접 설명하고 동의서를 온라인 서명 프로그램을 통해 참여자의 동의 서명을 받도록 한다.

### - 동의서 및 설명문

### ① 동의서 및 설명문 포함 내용 : 다음과 같은 사항을 포함하되, 각 사항에 대하여 연구책임자 또는 권한을 위임 받은 연구담당자는 연구참여자에게 문서를 읽고 설명할 기회를 제공한다. 또한 연구참여자의 질문에 대하여 만족할 만한 답변을 제공하며, 필요에 따라 연구참여자와 해당 연구에 대한 토의를 시행한다.

### - 연구과제명

### - 모든 과정은 연구 목적으로 수행된다는 사실

### - 본 연구의 배경과 목적

### - 본 연구의 참여자 선정 기준

### - 본 연구의 방법

### - 본 연구의 예상 참여 기간

### - 본 연구에 참여함에 따른 이익

### - 본 연구가 연구참여자에게 미칠 수 있는 부작용 또는 위험과 불편함

### - 본 연구에 참여함에 따른 보상 또는 비용

### - 본 연구의 개인정보 수집내용 및 보호대책. 모니터요원, 점검 요원, 기관생명윤리위원회는 연구참여자의 비밀보장을 침해하지 않고 관련규정이 정하는 범위 안에서 연구의 실시 절차와 자료의 신뢰성을 검증하기 위해 연구 결과를 직접 열람할 수 있다는 사실과 동의서 서식에 서명함으로써 연구참여자 또는 대리인이 이러한 자료의 직접 열람을 허용함을 의미한다는 사실

### - 연구참여자의 연구 참여 여부 결정은 자발적인 것이며, 연구에 참여하지 않을 경우 발생하는 불이익은 없다는 사실. 또한 연구 도중 언제라도 중도에 참여를 포기할 수 있으며 참여 중지 시 개인정보는 연구에 사용되지 않고 영구 삭제된다는 사실.

### - 연구와 연구참여자의 권익에 관해 추가적인 정보를 얻고자 하거나 연구와 관련이 있는 손상이 발생한 경우에 접촉해야 하는 사람

### ② 동의서 및 설명문 서명 과정 : 연구참여자가 자발적으로 연구 참여에 동의하도록 하며, 연구참여자 또는 법적 대리인이 연구 참여에 동의하지 않는다면 거절할 권리를 존중하도록 한다.

### - 연구 절차의 시작 및 등록 전 각 연구참여자나 그 법적 대리인으로부터 연구참여자의 동의서를 받는다. 이러한 책임은 연구참여자 동의서에 대한 적절한 설명을 제공한 후 연구참여자 서명 및 서명날짜를 받는 것을 포함한다.

### - 연구책임자 또는 위임 받은 연구담당자는 동의서에 서명 및 날짜를 기록한다.

### - 설명문을 포함하여 동의서 1부를 복사하여 원본은 연구책임자가 보관하고, 사본은 연구참여자에게 제공한다.

### - 각 연구자는 연구참여자를 등록시키기 전에 검토와 승인을 위하여 IRB에 연구참여자 동의서를 반드시 제출하고 승인된 동의서를 가지고 연구를 진행한다.

### - 만약 연구계획서에 변경이 생겨 변경된 정보를 제공해야 할 경우, 연구담당자는 변경된 동의서의 정보를 연구참여자에게 설명 후 해당 동의서를 제공하며, 서명된 동의서는 원본을 보관하고 사본은 역시 연구참여자에게 제공한다.

### ③ 동의서 설명 연구원 : 윤인애, 하지수, 김선중

1. **연구 방법(자료 수집 방법)**

### □ 일대일 in-depth interview 또는 focus group interview 방식을 통한 반구조화 된 표준적인 개방형 인터뷰 : Semi-structured open ended Interviews

### □ 물리적 거리 한계를 극복하기 위해 필요시 온라인 심층면담(ex. Zoom online meeting 프로그램 이용)을 사용할 수 있다.

### □ 반구조화 된 표준적인 개방형 인터뷰 : Semi-structured open ended Interviews

### - 장점 : 인터뷰에 참여하는 패널들이 어느 정도 정해진 틀 안에서 자유롭게 본인의 의사를 표현할 수 있게 해준다. 면담 형식 중 researcher bias 가 가장 적게 개입하여 이와 같은 질적 연구 수행 시 가장 많이 쓰이는 면담 방식.

### - 단점 : 나올 수 있는 답변의 종류 및 유형이 자유롭기 때문에 분석해야 하는 양이 방대해질 수 있으며 비슷한 주제별로 묶어서 코딩 분석하기 어려움이 있다.

### □ in-depth interview

### - 장점 : 1명 또는 소수의 연구참여자와의 상호작용을 통해 깊이 있는 자료 수집을 가능하게 해준다. 특정 현상에 대하여 개인적인 경험을 자세히 공유할 수 있고, 연구참여자의 응답에 기반하여 유연한 인터뷰 질문이 가능해 더 많은 정보를 얻을 수 있다.

### - 단점 : 개별 인터뷰로 시간과 비용이 많이 들고, 연구자의 의견이나 질문 방식에따라 참여자의 응답이 영향을 받아 편향될 수 있다

### □ focus group interview

### - 장점 : 다수의 연구 참여자들이 서로의 경험을 공유하고 의견을 낼 수 있어 다양한 관점과 새로운 통찰을 얻을 수 있다. 다양한 참여자들의 의견으로, 이는 연구 결과의 다양성과 일반화에 도움이 된다.

### - 단점 : 다양한 참여자들 속에서 개인의 의견을 자유롭게 표현하지 못하는 경우가 있을 수 있고, 다수의 의견에 영향을 받을 수 있다.

### - 인터뷰 장소는 연구 참여자가가 가능한 긴장하지 않고 편안함을 느낄 수 있는 조용하고 독립적인 장소를 섭외하여 진행한다.

### - 대면인터뷰가 불가능할 경우, Zoom이나 Google meeting과 같은 화상회의프로그램을 활용하여 진행될 수 있으며, 화상회의프로그램을 통해 설명문을 보면서 연구자가 직접 설명하고 동의서를 온라인 서명 프로그램을 통해 참여자의 동의 서명을 받도록 한다. 화상회의프로그램을 이용하여 온라인으로 인터뷰를 진행하는 경우에는 영상과 음성을 동시에 기록한다. 영상을 기록하더라도 보관 시에는 음성 기록만을 남길 것이며, 대상자가 영상 기록을 원치 않으면 화면 녹화 없이 음성으로만 인터뷰를 진행할 것이다. 이 내용을 인터뷰 일정 및 장소 조율 시에 충분히 설명하고 대상자가 대면 또는 온라인 인터뷰 방식을 선택할 수 있도록 한다.

### - 인터뷰 시간은 60분~ 150분 이내에 진행을 할 예정이다.

### - 자료 수집은 자료가 충분성과 적절성을 갖추고 포화상태에 이르렀을 때 종료할 것이다.

### - 익명으로 처리된다는 것을 밝히고 연구 대상자의 동의하여 인터뷰 내용을 녹음, 녹화를 실시할 것이다.

### - 인터뷰 내용은 녹음하고 면담 시 음성으로는 놓칠 수 있는 연구 참여자의 표정, 특징적인 행동이나 기타 사항을 메모하여 분석 시 참고하도록 한다.

1. **수집 항목**

### - 총 5단계의 인터뷰 과정을 통해 각 단계의 주제와 관련된 항목을 수집할 것이며 세부 주제 및 수집항목은 아래 표와 같다.

###

| **단계** | **주제** | **수집항목** |
| --- | --- | --- |
| **1.소개** | **참여자 소개** | **간단하게 자기 소개 부탁드립니다.**  **(나이/ 성별/ 근무지역/ 근무형태/ 현장근무기간/ 연구참여동기)** |
| **2.도입** | **전반적인 의료 이용에 대한 경험** | **평상시에 건강상태는 어떠한지?**  **주로 어떤 이유로 병원을 찾게 되는지?**  **병원을 선택하는 기준은 어떤 것인지?**  **그 경험에서 느낀 바는 무엇인지?** |
|  | **한의치료에 대한 인식** | **평소 한의치료에 대해 어떻게 생각하는지?**  **한의치료를 선호 또는 선호하지 않는지? 또 그 이유는?** |
|  | **한의치료에 대한 경험** | **한의치료에 대한 경험이 있는지?**  **있다면 어떤 증상으로 치료받았는지**  **한의치료를 받고 평소 인식에 변화가 있었는지?**  **한의치료를 받고 만족했는지? 만족/불만족 했다면 그 이유는 무엇인지?**  **경험이 없다면, 그 이유는?**  **한의치료의 어떤 점이 개선된다면 받을 의향이 있는지?** |
| **3.본론** | **국립소방병원 내 한의과 설치와 관련한 내용** | **국립소방병원의 설립을 눈앞에 두고 있는데 한의과 설치는 미정이다. 이에 대한 본인의 생각은 어떠한가?**  **한의과 설치/반대하는가? 그 이유는?** |
|  | **국립소방병원 내 한의과 이용에 대한 내용** | **국립소방병원 내 한의과가 설치된다면 이용할 의향이 있는지? 그 이유는?**  **어떤 증상에 어떤 한의치료 서비스를 이용할 것인지?** |
| **4.요약** | **인터뷰 내용 요약** | **앞선 인터뷰 내용을 요약 후 참여자의 의도대로 이해한 것이 맞는지 확인 받음.** |
| **5.끝맺음** | **추가적 내용 질의** | **추가적으로 언급하고 싶은 부분이 있는지?** |

**7) 효과 평가 기준 및 방법**

□ 자료 분석 과정

- 본 연구의 자료 분석은 Krippendorff(2003)가 제안한 내용분석(Content analysis) 절차에 따라 분석할 예정이다.

- 자료 분석 소프트웨어로는 Nvivo12 PLUS software를 구매하여 질적 연구 자료의 분석에 사용할 예정이다.

| \| 참여자들에게 연구의 목적과 내용을 설명하고 사전 동의를 받은 후  반구조화 된 질문지를 사용하여 심층면담 진행 \| \| --- \| \| ↓ \| \| 인터뷰의 모든 내용을 녹음하고 연구 보조자가 인터뷰를 관찰하여  특이사항 기록 \| \| ↓ \| \| 토론 내용이 제대로 기록되었는지 요약정리 된 내용을 확인하고  인터뷰 직후 연구진이 디브리핑(debriefing)시간을 가짐 \| \| ↓ \| \| 녹음한 내용을 즉시 전사하여 내용분석을 위한 준비 \| |
| --- | --- | --- | --- | --- | --- | --- | --- |

- 내용분석법은 코딩을 통해 데이터를 주관적으로 해석할 때 사용되는 연구방법이다. 이 분석법은 다른 질적 연구방법과는 달리 특정 연구방법론이나 철학적 가정을 필요로 하지 않는다. 또한 결과적으로 이론을 형성하지 않으며 단지 어떤 상황에 대한 이해를 목표로 한다. 본 연구에서는 결론을 통해 어떤 이론을 제시하기 보다는 어떤 현상을 이해하는 것을 목적으로 하므로, 내용분석법을 채택하였다.

- Krippendorff(2003)에 따른 분석방법은 다음과 같다.

첫째, 텍스트 전체에 대한 연구자의 이해과정으로 연구자들은 심층면담 기록을 반복하여 읽으면서, 연구참여자들의 의료이용경험이 소방공무원들의 한의치료에 대한 인식과 필요성에 관련하여 어떠한 관계가 있는지를 검토한다.

둘째, 의미 있는 진술(significant statement)을 찾는 과정으로 연구자는 참여자들이 구술한 내용을 문장으로 만들고, 이 문장들 중에서 한의치료와 관련된 의미 있는 진술들을 발견하여 의미를 재구성한다.

셋째, 범주화 단계이다. 연구자는 의미 있는 진술을 개념화한 후 이러한 개념들 중에서 상호 관계가 있거나 유사한 것들을 결집하여 범주로 구성한다.

넷째, 차원화 단계로, 구성된 범주를 사회적 차원, 개인적 차원, 인지적 차원으로 나누어 범주를 재배열한다.

- 연구자들은 자료수집과 분석에 있어서 충분성과 적절성을 충분히 고려한다. 충분성은 포화의 원칙에 입각하여 더 이상 새로운 개념이 출현할 수 없다고 판단될 때 자료 수집과 분석을 종료한다. 적절성은 인터뷰의 질문내용이 중요하다고 판단하여 반구조화 설문지를 개발하고 인터뷰의 진행 및 과정에 맞게 수정하고 보완하여 적절성을 확보한다.

□ 분석팀

- 합의팀 : 인터뷰를 진행한 한의사 2인 및 질적 연구 경험이 있는 한의약 임상연구 전공 교수1인, 인터뷰를 수행한 연구원 2인

- 감수팀 : 질적연구 경험이 많은 교수 2인

□ 연구의 타당도 확보

- Traiangulation(삼각 검증) : 비언어적 표현, 녹음등의 비교를 통해 시행한다.

- 주 인터뷰어의 코딩을 독립된 연구자가 타당성 확인

- COREQ 가이드라인에 기반하여 연구 보고의 질을 높임

- 코딩 : 상세 코딩 방법은 효과평가 기준 및 방법에서 설명함

- 전사자료의 확인 : 연구 대상자 중 EXCEL로 난수표를 만들어 무작위로 선정하여 확인

- 분석자료의 확인 : 연구 대상자 중 EXCEL로 난수표를 만들어 무작위로 선정하여 확인

1. **통계 분석 방법**

- 본 연구는 질적 연구이므로 자료의 분석은 코딩의 방법을 따르며 별도의 정량적 통계분석은 이뤄지지 않는다.

- 유사한 의미를 갖는 텍스트 자료를 범주화하여 코딩을 통해 주제나 패턴을 확인한다.

- 반복 청취 및 독해를 통해 주요 개념을 도출하고 명명한 후 서로 관련성이 있는 개념들을 하위범주로 분류한 후, 하위 범주의 관련성을 고려하여 범주로 추상화한다.

- Researcher bias를 최소화하기 위해 면담을 실시한 연구담당자를 제외한 연구진 내의 추가 2인이 독립적으로 평가하고 회의를 통해 확인하도록 한다.

- 질적연구 보고 시 표준 가이드라인으로 쓰이는 Consolidated criteria for reporting qualitative studies (COREQ) 항목에 맞춰서 결과를 보고한다.

1. **이익 및 위험**
   1. **연구의 이익 및 위험**

- 심층면담 따른 신체적, 정신적 피로 등이 예상됨.

- 연구에 참여함으로써 대상자 개인에게 돌아가는 직접적인 이익과 위험은 없으나 심층 면담을 통해 소방공무원의 한의치료의 경험 및 필요성 등에 대한 의견을 제공함으로써 국립소방병원 내 한의과 설치의 근거마련과 후속연구에 기초자료로 도움이 될 수 있음.

- 1. **연구대상자 안전대책, 보상 방안**

- 심층 면담 조사 로 연구와 관련 손상은 발생할 위험이 없으나 인터뷰 중간에 피로를 호소하면 중단하고 참여자가 연구 참여 철회를 원하면 즉시 연구를 중단시킨다.

1. **연구대상자 제공 사항**

- 심층 면담 이후 면담 참여에 대하여 1인 1회당 10만원(최소 1회, 최대 5회)의 사례비 제공

- 보상주체 : 연구비에서 지급

1. **연구대상자의 안전 보호를 위한 대책**
2. **연구의 윤리성 확보를 위한 기본 방안**

- 모든 과정에서 헬싱키 선언(64th WMA General Assembly, Fortaleza, Brazil, October 2013)을 준수할 것이다.

- 본 연구는 기관생명윤리위원회의 승인 후 수행할 것이며, 연구 수행 과정에서 일어나는 변경사항 등에 대하여 관련 규정을 준수할 것이다.

1. **연구대상자의 취약성 및 보호대책**

- 본 연구는 취약한 대상자를 대상으로 하지 않는다.

1. **연구대상자 모집 및 동의서 취득 시 대상자 보호 방법**

- 연구의 목적과 방법을 설명한 후 연구 참여에 동의하는 사람에 한해 서면 동의를 받고 연구 참여자로 선정한다.

- 면담을 통해 자료를 수집할 것이며 면담 내용은 메모 및 녹음할 것이라는 것을 사전에 알려주며 동의를 구한다.

- 연구 참여자의 사생활 보호 및 연구 자료의 기밀 유지를 위해 모든 자료는 이중 시건장치가 된 캐비닛이나 전자자료의 경우는 접근이 제한된 컴퓨터에 암호를 걸어서 보관할 것이며 연구 참여자는 식별정보로 코드화하고, 면담 자료는 연구 목적으로만 사용할 것이다. 이러한 사실을 연구 참여자에게도 알려준다.

- 연구 참여자의 자유의사에 따라 연구 참여를 중단할 수 있음을 알려 주고 연구 참여자에게 연구와 관련된 문이 사항을 언제든지 물어볼 수 있도록 관련 담당자 및 연락처를 제공한다.

1. **연구대상자의 개인정보보호 방안**

- 증례기록지 등 연구와 관련된 모든 서류에는 연구 참여자 이름이 아닌 연구대상 식별코드로 기록하고 구분한다. 논문에 개인을 식별할 수 있는 모든 정보(성명, 주민번호, 주소, 전화번호 등)는 기입하지 않고 개인정보 보호에 최선을 다할 것이다.

- 연구대상자의 신원을 파악할 수 있는 기록은 비밀로 보장될 것이다. 개인정보에서 보상비 지급을 위한 성명, 주소, 계좌번호, 이메일, 연락처 외의 정보는 수집하지 않으며 민감정보는 수집하지 않는다. 연구의 결과가 출판될 경우에도 연구참여자의 신원을 비밀상태로 유지한다.

- 연구 데이터는 잠금장치가 있는 캐비넷에 보관하고, 접근의 권한이 있는 연구자만 접근할 수 있다. 연구 데이터가 포함된 모든 문서는 암호로만 접근할 수 있도록 보안 설정을 하여 잠금장치가 있는 연구실 내 별도의 암호로만 접근할 수 있는 컴퓨터 저장장치에 보관하여 인증되지 않은 접근으로부터 기록을 보호할 것이다.

- 녹음 파일, 녹취 및 필사 자료는 잠금장치가 있는 연구실 내 암호가 설정된 컴퓨터 저장장치 안에 암호화되어 보관되며 연구가 종료된 시점으로부터 3년 후에 폐기될 것이다.

- 생명윤리법 시행규칙 제15조에 따라 연구 관련 기록은 연구가 종료된 시점부터 3년간 보관할 것이다. 보관기관이 지난 문서는 개인정보보호법 시행령 제16조에 따라 파기할 것이다.

1. **연구대상자의 중도 탈락 혹은 참여 철회 후의 자료의 처리 방법**

- 심층 면담 조사 연구 참여에 동의한 사람이 중도 탈락 혹은 연구 참여에 대한 동의를 철회하는 경우 그 즉시 연구에서 제외하며 데이터를 추가적으로 수집하지 않고, 해당 참여자의 녹음, 전사자료는 연구에 활용되지 않는다. 기수집된 데이터는 해당 참여자의 전사기록은 붉은색으로 처리하여 연구에 활용하지 않고 관련 법률에 따라 3년 보관 후 파기할 예정이다.

1. **참고문헌**
   1. Qaseem A, Wilt T, Mclean RM, Forciea MA, et al. Noninvasive Treatments for Acute, Subacute, and Chronic Low Back Pain: A Clinical Practice Guideline From the American College of Physicians. Ann Intern Med. 166(7): 514-530.2017
   2. 김상호, 권찬영, 서주희. 재난트라우마의 한의사 진료 매뉴얼. ㈜집문당. 2022
   3. Kang BS, Sul JU, Jeon SH, Cheon SH, Lemm JT, Jo SJ. Utilization of Acupuncture and Herbal Ointment Instead of Skin Graft Surgery for the Treatment of Burn Injuries: A Case Series and Literature Review. J Burn Care Res. 43(4):852-862. 2022
   4. Fernandez-Jane C. Vilaro J. Fei Y. Wang C. Liu J. et al. Acupuncture techniques for COPD: a systemic review. BMC Complement Med Ther. 20(1): 138. 2020
   5. King HC, Moore C, Spence DL. Exploring self-reported benefits of auricular acupuncture among veterans with posttraumatic stress disorder. J of holistic nursing. 34(3):291-299. 2016
   6. King HC, Spence DL, Hickey AH, Sargent P, et al. Auricular acupuncture for sleep disturbance in veterans with posttraumatic stress disorder: a feasibility study. Mil med. 180:582-590.2015
   7. Zeliadt SB, Thomas ER, Olson J,Coggeshall S, et al. Patient feedback on the effectiveness of auricular acupuncture on pain in routine clinical care. Med care,58: 101-107. 2020
   8. Kligler B, Niemtzow RC, Drake DF, Ezeji-Okoye SC, et al. The Current State of Integrative Medicine Within the U.S. Department of Veterans Affairs. Med Acupunct. 30(5): 230-234. 2018
   9. Farmer MM, McGowan M, Yuan AH, Whitehead AM, et al. Complementary and integreative health approaches offered in the veterans health administration: Results of a national organizational survey. J Altrn Complement Med. 27(S1):S124-S130. 202
   10. 부산대학교 한의학전문대학원. 국공립병원 내 한의 공공의료 확대 방안 연구. 한국한의약진흥원. 2021.
   11. Krippendorff, K. (2003). Content Analysis: An Introduction to Its Methodology, (2nd ed.), Thousand Oaks, CA: Sage publications, Inc.
