# Supplementary material for: Firefighters’ medical use and Korean Medicine experience in Korea: A qualitative study protocol
Source: PLoS One. 2024 Mar 25;19(3):e0300532. doi: 10.1371/journal.pone.0300532 (PMC10962829; doi:10.1371/journal.pone.0300532)
Supplement: S2 File — (DOCX) [file pone.0300532.s003.docx]

**Research Plan**

**Qualitative Study on the Perception and Demand for Overall Medical Utilization and Korean Medicine Treatment among Firefighters**

**Version No: Ver.1.2.**

**Institution: Korean Medicine Department, National Medical Center**

**Chief researcher: In-ae Yoon**

**Thesis: Yes ( o ) / No ( ) Research Plan**

1. **Title**

Qualitative Study on the Perception and Demand for Overall Medical Utilization and Korean Medicine Treatment among Firefighters

1. **Name and address of the institution**

### National Medical Center, 245 Eulji-ro, Jung-gu, Seoul (18-79, Eulji-ro 6-ga)

1. **Name and position of the research director and co-researchers**
2. **Chief researcher**

### In-ae Yoon (Director of Korean Medicine & Acupuncture Department, National Medical Center)

1. **Co-researchers**

### Jung-tae Lim (Professor of College of Korean Medicine, Wonkwang University)

Jin-won Kim (Head of the Korean Medicine Department, National Medical Center)

### Ji-su Ha (Resident of the Acupuncture Department, National Medical Center)

### Sun-jung Kim (Resident of Acupuncture Department, National Medical Center)

1. **Name and address of the research funding institution**

### National Research Foundation of Korea

Government complex in Daejeon: Gajung-ro 201, Yuseong-gu, Daejeon

Government complex in Seoul: 25 Heonneung-ro, Seocho-gu, Seoul

1. **Estimated research period**

### IRB approval date to December 31, 2024

1. **Target of research**

### National firefighting officers

1. **Background and purpose of research**
2. **Background**

### □ Establishment of the National Fire Hospital

### - Firefighting officers, due to the nature of their duty, are always exposed to dangerous, gruesome, and traumatic situations at disaster sites, and they suffer from mental pain such as injuries and trauma. Therefore, systematic treatment, research, and management are required.

### - Accordingly, facilities and medical staff that specialize in treating, managing, and researching diseases related to firefighting officers are needed.

### - The Act on the Establishment and Operation of the National Fire Hospital will be enacted and implemented, and the hospital will be established for systematic health care and education of firefighting officers through the treatment of firefighting officers, analysis of harmful health factors caused by special working conditions, and disease research.

### □ Necessity of the establishment and operation of the Korean medicine department in the National Fire Hospital

### ○ Carrying out the function and role of a national public medical institution

### - Establishment is necessary to ensure the medical options and accessibility of the public as a public medical institution

### ○ There is a close relationship between the health problems experienced by firefighting officers and Korean medicine.

### - Among the 10 prevalent medical conditions of firefighting officers (health insurance), 6 are found in the frequent diseases in Korean medicine.

### - Musculoskeletal diseases, which have a high prevalence among firefighting officers, are a representative field of treatment for Korean medicine, and 18 of them are included in the 50 most frequent diseases in Korean medicine.

### 2) Previous studies

### □ A previous study on Korean medicine treatment related to diseases and injuries caused by the special working environment of firefighting officers

### - Korean medicine treatment for Musculoskeletal pain

### American College of Physicians, the second largest medical group in the US, strongly recommended acupuncture treatment for acute/subacute back pain (low-quality evidence), chronic back pain (moderate-quality evidence), and Chuna Manual Therapy (spinal manipulation, low-quality evidence) in the guideline for noninvasive care of acute, subacute, and chronic back pain.

### - Korean medicine treatment related to PTSD

### Based on several studies with the support of the National Research Foundation of Korea, a manual for Korean medicine treatment for disaster trauma was published in 2022.

### - Korean medicine treatment for burns

### A case report of four patients who suffered deep burns requiring skin transplants and significantly improved their condition through herbal medicine ointment and acupuncture without surgery was published in an international journal of treatments for burns.

### - Research on the respiratory system

### As a result of reviewing 33 studies in the literature review on acupuncture treatment for chronic obstructive pulmonary disease, acupressure treatment was found to be effective in relieving dyspnea, improving quality of life, and reducing anxiety.

### 3) The need for research

### □ Overseas cases (Public medical institutions for veterans in the US)

### ○ Previous studies on Korean medicine treatment for veterans in the US

### - Clinical studies of Korean medicine treatment for veterans suffering from diseases/injuries due to special working conditions, such as firefighting officers, demonstrate the effectiveness of Korean medicine treatment on PTSD, insomnia caused by PTSD, and pain of veterans.

### ○ Rise of veterans’ demand and expansion of medical services for integrated medical care (including Korean medicine treatment).

### - The demand for integrated medical care is increasing as an alternative to the opioid crisis of drug treatment for chronic pain, anxiety, depression, PTSD, and addiction.

### - A study on the demand for Korean medicine treatment for soldiers showed a high rate of veterans’ demand for and desire to use acupuncture treatment (a survey of 3,346 soldiers showed that more than 84% had experience in using integrated medicine).

### - Accordingly, public medical institutions for veterans in the US intend to expand and provide Korean medicine services.

### ○ In addition, The Veterans Health Administration (VA) of the US mandated that the provision of integrated medical approaches, including acupuncture and Chuna Manual Therapy, be expanded through legislation in 2016 (2016 Comprehensive Addiction and Recovery Act).

### □ Currently, the establishment of the Korean medicine department in the National Fire Hospital, which is scheduled to open in June 2025, is undecided.

### - As described above, Korean medicine services can play a significant role in providing treatment for diseases and injuries caused by the special working conditions of firefighting officers, and there are many research results on their effects.

### - Internationally, the establishment of a Korean medicine department in public medical institutions in consideration of preference and effectiveness is becoming mandatory, while in Korea, there is no plan to establish a Korean medicine department in the newly established National Fire Hospital even though the country has the most personnel for Korean medicine treatment.

### - According to a study on expansion measures for a Korean medicine department in public medical institutions, discussing the matter is more difficult in existing public hospitals. It is, rather, more efficient to discuss the issue before the establishment of a medical institution is completed.

### - Therefore, at this point where the establishment of the National Fire Hospital, which should provide appropriate medical treatment and best medical services to firefighting officers and conduct related research, is imminent, it is necessary to discuss the establishment of the Korean Medicine Department.

1. **Research hypothesis and purpose**

□ The purpose of this study is to investigate the preference, demand, satisfaction, and overall perception and experience of Korean medicine treatment of firefighting officers and study the feasibility and necessity of the establishment of the Korean medicine department in the National Fire Hospital.

□ Furthermore, the results of this study will be used as evidence for the establishment of the Korean medicine department in public hospitals, data for related policies, and as an exemplary model for subsequent related cases.

1. **Research method**
2. **Selection and exclusion criteria of research participants**

**- Selection criteria**:

- - - - 19 to 65 years of age
      - A person who is currently or has experience as a firefighting officer on-site
      - A person who meets the above conditions and voluntarily agrees to participate in the research after receiving sufficient explanation of the research

**- Exclusion criteria:**

- A firefighting officer who has no experience on-site
- If the researcher determines that conducting an interview is impossible

**- Dropout criteria:** If the participant no longer is willing to proceed with the study

1. **Number of participants**

- Number of participants for an in-depth interview: 20 firefighting officers

- This includes the participants for both a one-on-one interview and a focus group interview. Participants who are included in both interviews are excluded from counting.

- The number of participants for the one-on-one interview and the focus group interview is unlimited.

1. **The basis for calculating the number of participants**

- The sampling of participants will end when data is saturated, and the validity of the number of participants will be secured based on Duke’s (1984) recommendation for conducting a qualitative study on experience (10 to 20 participants) and the sampling number suggested by other qualitative studies on firefighting officers. However, additional interviews may be conducted if the data is not saturated after 20 participants have been interviewed.

- To obtain a balanced perspective, there shall be an equal number of participants who have experience in Korean medicine treatment and those who do not.

- The focus group interview consists of 6 to 10 participants per group, and the participants can be selected among those who are one-on-one interviewees, or a separate panel can be formed.

- Saturation refers to a state in which no longer a new story is derived from a new panel and the story mentioned in the existing panel is repeated, making it unnecessary to perform a qualitative interview of the next panel.

1. **Recruitment method of participants and consent process**

### □ Recruitment of participants

### - Recruitment is scheduled to commence on October 4, 2023, and conclude on December 31, 2024.

### - After requesting cooperation from fire departments in an official letter using convenience sampling, research and website advertisement links will be posted on the intranet for firefighting officers. The advertisements shall be posted after IRB approval containing the research title and content, the criteria for research participants, the date and time of the interview, the location, the benefits and disadvantages of participating, and the contact information of the researcher.

### - Moreover, research participants will be recruited through snowball sampling, in which new participants are introduced by previously recruited participants.

### □ Consent of the participants

### - Consent process: Research participants are reviewed for suitability for the study. Then, the research director in charge presents the manual and consent form that includes all matters related to the consent required by the relevant laws and regulations and explains the contents in writing to the research participants or the legal representative.

### - If it is difficult for the participant to have a face-to-face interview due to time and place constraints, the researcher shall explain the descriptions directly through a video conference (Zoom or Google Meet) and obtain the participant’s consent through an online signature program.

### - Consent and description forms

### ①Details of the consent and description forms: The following matters shall be included, and the research director or delegated research manager shall provide the participant the opportunity to read and request an explanation of the documents. Satisfactory answers to the participant’s questions shall be provided, and discussions on the research shall be conducted as necessary.

### - Research project title

### - The fact that all processes are carried out for research purposes.

### - Background and purpose of the study

### - Selection criteria of participants

### - Research method

### - Estimated period of participation

### - Benefits of participating in the study

### - Possible side effects, risks, or inconveniences due to participation

### - Compensation or costs of participation

### - Collection of personal information and protection measures of the study. The fact that monitoring personnel, inspection personnel, and institutional bioethics committees may directly access research results to verify the reliability of data and research procedures without infringing on the confidentiality of the research participants, and that signing a consent form means that the research participant or the representative concedes to the direct access of such data.

### - The fact that the research participant’s decision to participate in the study is voluntary, and there is no disadvantage of not participating. Moreover, the participant may drop out at any time during the research, and personal information shall be permanently deleted without being used in case of a dropout.

### - The person to contact in case research participants desire to obtain additional information about the research and their rights and interests, or in case damage has occurred related to the research.

### ② Signing of documents: The research participant should voluntarily agree to participate in the study, and the right to refuse participation in the study must be respected.

### - The consent from each research participant or their legal representative shall be obtained prior to the start of research and registration. The research participant’s signature and signature date must be included in the consent after providing an appropriate explanation for the participant’s consent.

### - The research director or delegated research manager shall record the signature and date on the consent form.

### - The research director keeps the original copy of the consent form, including the descriptions, and provides a copy to the research participant.

### - Each researcher must submit a research participant’s consent to the IRB for review and approval before registering the research participant and conducting the study with the approved consent.

### - If changes occur in the research plan and the information needs to be provided, the research director shall provide a revised consent form to the research participant after explaining the changes. The research director keeps the original signed consent and provides a copy to the participant.

### ③ Researchers who will provide an explanation regarding consent: In-ae Yoon, Ji-soo Ha, and Sun-joong, Kim

1. **Research method (Data collection)**

### □ Semi-structured open-ended Interviews through one-on-one in-depth interviews or focus group interviews

### □ In-depth online interviews (e.g., Zoom online meeting) may be used if necessary to overcome limitations due to physical distance.

### □ Semi-structured open-ended Interviews:

### - Advantage: The panelists participating in the interview can freely express their opinions within a certain framework. Research bias intervenes the least among interview methods, and thus is most commonly used for conducting qualitative research.

### - Disadvantage: The amount of data for analysis will be vast due to unlimited answer types, and it will be difficult to conduct code analysis by categorizing similar topics into groups.

### □ In-depth interview

### - Advantage: It enables in-depth data collection through interaction with one or a small number of research participants. Research participants can share personal experiences about specific phenomena in detail, and flexible interview questions can be asked based on the participants’ responses.

### - Disadvantage: Individual interviews are time-consuming and expensive, and the participant’s response may be biased due to the researcher’s opinion or questioning method.

### □ Focus group interview

### - Advantage: Various perspectives and new insights can be gained as multiple participants share their experiences and express opinions with each other. Opinions of various participants will benefit the diversity and generalization of the study results.

### - Disadvantage: Some individuals may not feel free to express their opinions among other participants, and they may be influenced by the opinion of the majority.

### - The interview must be conducted in a quiet and independent place where the participants feel comfortable.

### - The interview may be conducted using video conference programs such as Zoom or Google Meet if face-to-face interview is impossible. The researcher shall directly provide an explanation through the video conference and obtain the consent form through an online signing program. Video and audio shall be recorded at the same time when conducting an online interview. For record-keeping, only the voice record shall be archived. If the participant is unwilling to video record, only a voice interview shall be conducted. Such information shall be fully explained when coordinating the interview schedule and location, allowing the participant to choose between a face-to-face and an online interview.

### - The interview period is between 60 to 150 minutes.

### - Data collection ends when the data reaches saturation with sufficiency and relevance.

### - The interview will be recorded in video and audio with the consent of the participants after revealing that the interview will be anonymously processed.

### - The facial expressions, characteristic behaviors, or other matters of the participants that may be missed by voice recording during the interview shall be written in notes for reference during analysis.

1. **Collection items**

### - Items related to the topics of each stage are collected through a five-stage interview. The detailed topics and collection items are shown in the table below.

###

| **Stage** | **Topic** | **Item** |
| --- | --- | --- |
| **1. Intro 1** | **Introduction of the participant** | **Briefly introduce yourself (age/gender/service area/work type/working career on-site/motive for participating in the study)** |
| **2. Intro 2** | **Overall experience with medical care** | **How is your normal health?**  **Normally, why do you visit the hospital?**  **What are the criteria for choosing a hospital?**  **How has been your experience of hospital visits?** |
|  | **Perception of Korean medicine treatment** | **What are your usual thoughts on Korean medicine treatment?**  **Do you prefer or not prefer Korean medicine treatment? And why?** |
|  | **Experience in Korean medicine treatment** | **Do you have any experience in Korean medicine treatment?**  **If so, what symptoms were you treated for?**  **Was there any change in your perception of Korean medicine treatment after receiving treatment?**  **Were you satisfied with Korean medicine treatment? What are the reasons you were satisfied/unsatisfied?**  **If you do not have experience, why?**  **What improvements in Korean medicine treatment should be made for you to be willing to receive the treatment?** |
| **3. Body** | **Related to the establishment of the Korean Medicine Department in the National Fire Hospital** | **We are on the threshold of establishing a National Fire Hospital, but whether a Korean medicine department will be set up is undecided. What is your opinion on this?**  **Do you agree/disagree with the establishment of the Korean medicine department? Why?** |
|  | **Information on the use of the Korean medicine department in the National Fire Hospital** | **Would you be willing to use the Korean medicine department if it were to be established in the National Fire Hospital? Why?**  **What kind of Korean medicine service will you use for what kind of symptoms?** |
| **4. Summary** | **Summary of interview** | **After summarizing the above interview, confirm that the participant’s responses were correctly understood.** |
| **5. Conclusion** | **Additional questions** | **Is there anything else you would like to mention?** |

**7) Criteria and method of efficacy evaluation**

□ Data analysis process

- The data will be analyzed according to content analysis proposed by Krippendorff (2003).

- For data analysis software, Nvivo12 PLUS software will be used to analyze qualitative research data.

| \| Conduct in-depth interviews using semi-structured questionnaires after explaining the purpose and content of the study to the participants and obtaining their prior consent. \| \| --- \| \| ↓ \| \| The research assistant takes notes of specifics after recording and observing all of the interviews \| \| ↓ \| \| Ensure the discussion is properly recorded and summarized. The researchers conduct a debriefing immediately after the interview. \| \| ↓ \| \| Immediately transcribe the recorded content and prepare for content analysis \| |
| --- | --- | --- | --- | --- | --- | --- | --- |

- Content analysis is a research method used to subjectively interpret data through coding. Unlike other qualitative research methods, this method does not require specific research methodologies or philosophical assumptions. Also, it does not form a theory as a conclusion but only aims to understand a certain situation. In this study, content analysis was adopted because the study aims to understand a certain phenomenon rather than present a theory through conclusions.

- The analysis method according to Krippendorff (2003) is as follows.

The first step is the researcher’s understanding of the entire text. As the researchers repeatedly read in-depth interview records, they examine how the research participants’ medical experience is related to the perception and necessity of Korean medicine treatment of firefighting officers.

The second step is the process of discovering significant statements. The researchers turn the participants’ oral statements into sentences, find significant statements related to Korean medicine treatment among the sentences, and reconstruct the meaning of those sentences.

Third, the categorization stage. After conceptualizing significant statements, the researchers gather interrelated or similar concepts and organize them into categories.

Fourth, the dimensionalization stage. The organized categories are divided into social, personal, and cognitive dimensions and are rearranged.

- The researchers shall fully consider the sufficiency and relevance of data collection and analysis. In terms of sufficiency, based on the principle of saturation, data collection and analysis end when it is judged that a new concept can no longer emerge. Relevance is secured by developing a semi-structured questionnaire based on the important interview questions and modifying and supplementing the questionnaire according to the progress and process of the interview.

□ Analysis team

- Consensus team: 2 Korean medicine doctors who conducted the interviews, 1 professor of clinical research of Korean medicine with qualitative research experience, and 2 researchers who conducted the interviews

- Supervision team: 2 professors with extensive experience in qualitative research

□ Securing the validity of the study

- Traiangulation: Performed through comparison of non-verbal expressions, recordings, etc.

- The coding of the main interviewer is validated by an independent researcher

- The quality of the research report is improved based on COREQ guidelines.

- Coding: Detailed coding methods are described in the criteria and methods for efficacy evaluation

- Confirmation of transcription data: A random number table is created with EXCEL and selected randomly among the study participants.

- Confirmation of analysis data: A random number table is created with EXCEL and selected randomly among the study participants.

1. **Analysis of statistics**

- Since this is a qualitative study, data analysis follows the coding method, and no separate quantitative statistical analysis is performed.

- Text data with similar meanings are categorized to identify topics or patterns through coding.

- After the main concepts are derived and named through repeated listening and comprehension, the concepts that are related to each other are classified into subcategories. Considering the relevance of the subcategories, they are then abstracted into categories.

- To minimize researcher bias, 2 additional researchers, excluding the research director who conducted the interviews, should evaluate the interviews independently and confirm them through a meeting.

- The results of the qualitative research shall be reported in accordance with the items in the consolidated criteria for reporting qualitative studies (COREQ), which is used as the standard guidelines.

1. **Benefits and risks**
   1. **Benefits and risks of the study**

- Physical and mental fatigue due to in-depth interviews is expected.

- There will be no direct benefit or risks to individual participants. By providing their experiences with and opinions on the necessity of Korean medicine treatment as firefighting officers through in-depth interviews, they can contribute to finding grounds to establish the Korean medicine department in the National Fire Hospital and provide basic data for follow-up research.

- 1. **Safety and comprehension measures for research participants**

- There is no risk of damage due to in-depth interviews related to the study. However, the interview should cease if the participant complains of fatigue and expresses the desire to withdraw participation from the study.

1. **Compensation to participants**

- A case fee of 100,000 won per person (minimum 1 time, maximum 5 times) is provided for in-depth interviews

- Agent: Paid from research funds

1. **Safety protection measures for research participants**
2. **Basic measures for securing research ethics**

- The Helsinki Declaration (64th WMA General Assembly, Fortaleza, Brazil, October 2013) shall be observed throughout the process.

- This study shall be conducted after approval by the IRB and the relevant regulations for changes that occur in the process of conducting research will be observed.

1. **Vulnerability and protection measures of research participants**

- This study does not target vulnerable subjects.

1. **How to protect research participants in the process of recruitment and obtaining consent**

- After explaining the purpose and method of the study, only those who agree to participate are selected as participants after obtaining written consent.

- Data is collected through interviews, and consent to take notes and record interviews is obtained in advance.

- To protect the privacy of research participants and to maintain the confidentiality of research data, all data shall be stored in a cabinet with dual locks, and all electronic data shall be stored on a restricted computer with a password. The research participants shall be coded with ID information, and interview data shall be used for research purposes only. Research participants shall be informed of the above facts.

- The participants shall be informed that they are free to terminate their participation in the study and be provided with related personnel and contact information to ask questions related to the study at any time.

1. **Measures to protect the personal information of research participants**

- All documents related to the study, such as case records, shall record and classify research participants with identification codes and their names. To protect personal information, the name, resident number, address, and phone number shall not be filled in the thesis.

- Records that verify the participant’s identity will be kept confidential. Information other than name, address, account number, e-mail, and contact number which are needed for compensation payments shall not be collected. Even after the study is published, the identity of the participants shall be kept confidential.

- Research data shall be stored in a locked cabinet and shall only be accessed by authorized researchers. All documents containing research data shall be stored in a computer storage device in the laboratory that can be accessed only with a separate password to protect records from unauthenticated access.

- Voice-recorded files, tape-recorded data, and transcribed data shall be stored in a computer storage device in a locked laboratory that can be accessed with a password and be discarded three years after the completion of the study.

- In accordance with Article 15 of the Enforcement Regulations of the Bioethics Act, research-related records will be stored for three years from the completion of the study. Expired documents will be destroyed in accordance with Article 16 of the Enforcement Decree of the Personal Information Protection Act.

1. **How to process data after dropout or withdrawal of participants**

- If a person who had agreed to participate in an in-depth interview drops out of the study or withdraws consent to participate, the participant shall be excluded from the study immediately and additional data shall not be collected. The participant’s recordings and transcription data shall not be used for research. The previously collected transcription data shall be processed in red and destroyed after three years according to relevant laws.

1. **Reference**
   1. Qaseem A, Wilt T, Mclean RM, Forciea MA, et al. Noninvasive Treatments for Acute, Subacute, and Chronic Low Back Pain: A Clinical Practice Guideline From the American College of Physicians. Ann Intern Med. 166(7): 514-530.2017
   2. Sang-ho Kim, Chan-young Kwon & Ju-hee Seo. *Disaster Trauma Manual for Korean Medicine Doctors*. Jipmundang Co., Ltd. 2022.
   3. Kang BS, Sul JU, Jeon SH, Cheon SH, Lemm JT, Jo SJ. Utilization of Acupuncture and Herbal Ointment Instead of Skin Graft Surgery for the Treatment of Burn Injuries: A Case Series and Literature Review. J Burn Care Res. 43(4):852-862. 2022
   4. Fernandez-Jane C. Vilaro J. Fei Y. Wang C. Liu J. et al. Acupuncture techniques for COPD: a systemic review. BMC Complement Med Ther. 20(1): 138. 2020
   5. King HC, Moore C, Spence DL. Exploring self-reported benefits of auricular acupuncture among veterans with posttraumatic stress disorder. J of holistic nursing. 34(3):291-299. 2016
   6. King HC, Spence DL, Hickey AH, Sargent P, et al. Auricular acupuncture for sleep disturbance in veterans with posttraumatic stress disorder: a feasibility study. Mil med. 180:582-590.2015
   7. Zeliadt SB, Thomas ER, Olson J,Coggeshall S, et al. Patient feedback on the effectiveness of auricular acupuncture on pain in routine clinical care. Med care,58: 101-107. 2020
   8. Kligler B, Niemtzow RC, Drake DF, Ezeji-Okoye SC, et al. The Current State of Integrative Medicine Within the U.S. Department of Veterans Affairs. Med Acupunct. 30(5): 230-234. 2018
   9. Farmer MM, McGowan M, Yuan AH, Whitehead AM, et al. Complementary and integreative health approaches offered in the veterans health administration: Results of a national organizational survey. J Altrn Complement Med. 27(S1):S124-S130. 202
   10. 2021. Busan National University Graduate School of Korean Medicine. *A Study on Measures to Expand Public Health Services through Korean medicine in National and Public Hospitals*. National Institute for Korean Medicine Development. 2021.
   11. Krippendorff, K. (2003). Content Analysis: An Introduction to Its Methodology, (2nd ed.), Thousand Oaks, CA: Sage publications, Inc.
